# Firing-Associated Recycling of Coal-Fired Power Plant Fly Ash

**DOI:** 10.1155/2023/8597376

**Published:** 2023-02-27

**Authors:** Vu Thi Ngoc Minh, Vuong-Hung Pham, Vu Hoang Tung, Cao Tho Tung, Nguyen Thi Hong Phuong

**Affiliations:** ^1^School of Chemical Engineering, Hanoi University of Science and Technology (HUST), No. 01, Dai Co Viet Road, Hanoi, Vietnam; ^2^Advanced Institute for Science and Technology (AIST), Hanoi University of Science and Technology (HUST), No. 01, Dai Co Viet Road, Hanoi, Vietnam

## Abstract

Coal-fired power plant fly ash is a global environmental concern due to its small particle size, heavy metal content, and increased emissions. Although widely used in concrete, geopolymer, and fly ash brick production, a large amount of fly ash remains in storage sites or is used in landfills due to inadequate raw material quality, resulting in a waste of a recoverable resource. Therefore, the ongoing need is to develop new methods for recycling fly ash. The present review differentiates the physiochemical properties of fly ash from two coal combustion processes: fluidized bed combustion and pulverized coal combustion. It then discusses applications that can consume fly ash without strict chemical requirements, focusing on firing-associated methods. Finally, the challenges and opportunities of fly ash recycling are discussed.

## 1. Introduction

Since the late 19th, coal-fired power has continuously grown and has been an essential source of global electricity. As of 2019, coal-fired power accounts for 36.7% of the world's total electricity production and 22.4% of the total electricity of countries in the organization for economic cooperation and development (OECD) [1]. Due to environmental concerns and the need for sustainable development, coal-fired power has gradually been replaced by other energy sources, such as wind, solar, natural gas, biomass, and combustible waste power. However, under the pressure of economic development, despite the decrease in proportion, the total capacity of coal-fired power continues to grow, especially in Asian countries such as China, India, and Southeast Asia, threatening net-zero targets by 2050. The IEA predicts that coal-fired power generation from 2021 to 2024 will increase by 4.1% in China, 11% in India, and 12% in Southeast Asia [2].

Environmental concerns arise from fuel extraction operations, the coal power generation process, and emission treatment. The primary discharge of coal combustion includes acidic gases such as SO_2_, NO_x_, and CO_2_; heavy metal vapors such as mercury; and solid combustion residues such as dust, fly ash, and bottom ash. Strongly acidic gases, including NO_x_ and SO_2_, go through chemical processes depending on the combustion system. Conventional boilers use low-ash coal grades and the combustion chamber temperature is usually above 1400°C. At this temperature, nitrogen gas oxidizes, forming NO_x_ in the flue gas. The flue gas is directed to an adsorption tower that performs selective catalytic reduction (SCR) to convert NO_x_ into nitrogen gas and water. The SO_2_ gas formed by the oxidation of sulfur-containing substances is absorbed in a wet flue gas desulfurization (WFGD) system with lime/limestone slurry to produce calcium sulfate. Fluidized bed combustion boilers often use high-ash coal grades and the temperature of the combustion chamber usually does not exceed 900°C to limit the generation of NO_x_. In this system, limestone can be fed into the combustion chamber to absorb SO_2_ gas [3].

Coal fly ash is also a primary environmental concern due to its small particle size and heavy metal contents. Continued increases in coal-fired power generation have resulted in a continued increase in global coal ash emissions. While a large amount of coal fly ash is used in nonfiring construction materials such as concrete [4–6], fly ash bricks [7, 8], and geopolymer [9, 10], a significant amount remains at the storage site or is used for landfills. A recent review of fly ash usage in China shows that 56% of fly ash is used in construction, 35% is used in landfills, and 9% is used in other applications [11].

For safety reasons, fly ash used as pozzolanic additives in concrete and cement has stringent quality requirements. The American standard ASTM C618-19 classifies fly ash used for concrete into classes F and C by chemical composition, loss-on-ignition, physical properties, and pozzolanic activity [12]. Chemically, the most significant difference between Class C and Class F is in the CaO content and the total SiO_2_ + Al_2_O_3_ + Fe_2_O_3_ content. Due to its high CaO content, often associated with lime and calcium sulfate, Class C fly ash features self-adhesive properties [13]. Class F fly ash contains a higher SiO_2_ content than Class C and shows pozzolanic activity in mortar and concrete. Ones that do not belong to Class C and Class F are not considered pozzolanic additives. Due to the quality requirements mentioned above, a large portion of fly ash piles up in storage sites by the power plant or is used for landfills and mine backfills [14, 15].

Under environmental stress, intensive studies have focused on thoroughly treating and utilizing fly ash in more value-added applications. In India, 32.87% of fly ash was left untreated during 2017–2018 [16], which decreased to 7.59% by 2020–2021 [17]. During the year 2020–2021, ten modes of fly ash utilization were effective: cement (25.81%), mine filling (6.20%), bricks and tiles (12.98%), reclamation of low-lying area (15.59%), ash dyke raising (7.94%), roads and flyovers (15.04%), agriculture (0.03%), concrete (0.83%), hydropower sector (0.03%), and others (7.97%) [17]. In this example, the main distribution of fly ash is in construction, of which the portion used for landfill and foundation reclamation accounts for a greater proportion than that used in cement, concrete, brick, and tiles. The environmental concerns associated with such applications include wind-blown dust [18] and the dissolution of toxic metals into groundwater [19, 20]. Therefore, methods of using fly ash to mitigate these risks are still under study.

Due to its porous structure, fly ash has a large specific surface area, which is suitable as a substrate in waste gas and wastewater treatment. In gas treatment, fly ash is mixed with calcium hydroxide to adsorb SO_2_ in flue gas [21, 22]. This mixture can also absorb toxic organic vapors such as toluene [23] and m-xylene [24]. In wastewater treatment, fly ash improves the precipitation of heavy metals [25], boron [26], and phosphate [27] in the presence of calcium hydroxide. In addition, fly ash can adsorb phenolic compounds [28, 29], dyes [30, 31], and pesticides [32]. Although economically and technically efficient to some extent, these applications accumulate toxic substances on the fly ash, making post-adsorption treatment of fly ash even more complicated.

Other applications of fly ash include raw materials for glass [33, 34], glass ceramics [35, 36], sintered bricks [37, 38], and ceramic tiles [39, 40]. This review covers the physical and chemical properties of coal fly ash and focuses on the firing-associated recycling of this material. The main objective of the review is to provide evidence that coal fly ash can be used as ceramic raw material without violating regulations on the release of heavy metals into the environment during use. The review also proves that this method can recycle a large amount of fly ash remaining in the storage sites because it does not place any limit on the origin and quality of fly ash.

## 2. Methodology

Most of the documents cited in this review paper are publications from Google Scholar and Web of Science databases. The statistical data from the International Energy Agency (IEA) and the India Central Electricity Authority (CEA) and test methods of the American Society for Testing and Materials (ASTM) and the International Standards Organization (ISO) are also cited.

As a supplement to the review, coal fly ash samples were collected and characterized. The samples were collected from four coal-fired power plants in Vietnam: Dong Trieu, Mong Duong I, Uong Bi, and Ninh Bình. Dong Trieu and Mong Duong I power plants apply the fluidized bed combustion (FBC) technique, whereas Uong Bi and Ninh Binh power plants employ the pulverized coal combustion (PCC) technique. The fly ash was dried overnight at 115°C before being characterized by X-ray diffractometry, scanning electron microscopy, and laser scattering particle size analysis.

A Bruker X-ray Diffractometer model D2 Phaser was used for phase analysis. Care was taken to keep the top surface of the sample mass flat and on the plane of the top of the sample holder to avoid possible displacement errors. The copper radiation source of the instrument was operated at 30 kV and 20 mA while scanning between 10 and 60 degrees 2-*θ*. The data collected from the diffractometer were analyzed using Crystal Impact's Match software using the COD database.

The microstructural properties of fly ash were investigated by a JOEL 6360LV scanning electron microscope. Fly ash samples were sprinkled onto PELCO Tabs™ carbon conductive tabs adhered to the SEM sample holders, which were then gently tapped to remove nonadherent particles. Before being placed in the SEM instrument, the sample holders were placed in a sputter coater to coat the fly ash particles with a thin layer of platinum for conductive purposes. The SEM ran under a vacuum.

The fly ash particle size distribution was analyzed using a HORIBA Partica Mini LA350 instrument, which used a laser source with a wavelength of 650 nm. The instrument works on the principle of laser diffraction and Mie's light scattering theory.

## 3. Properties of Coal-Fired Power Plant Fly Ash

**Figure 1:**
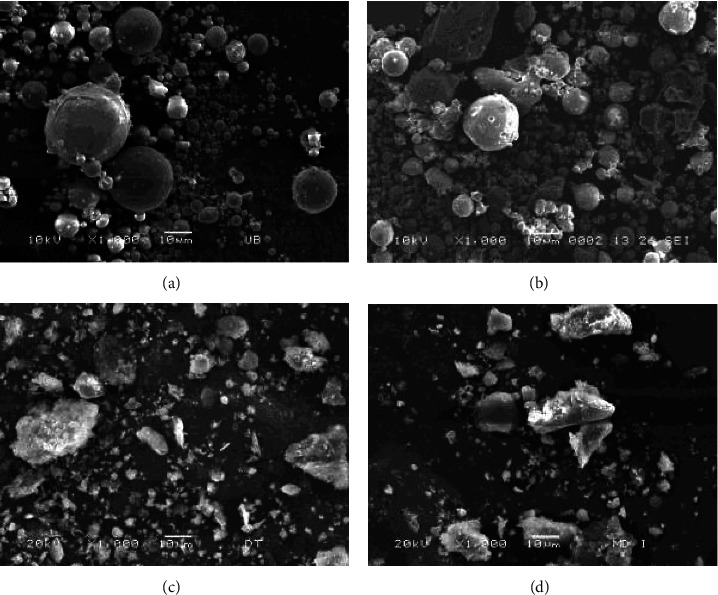
Scanning electron microscopy images of fly ash: (a) Uong Bi PCC, (b) Ninh Binh PCC, (c) Dong Trieu FBC, and (b) Mong Duong I FBC.

Coal combustion techniques significantly influence the morphology, particle shape, and particle size distribution of fly ash.  Figure 1 presents the scanning electron microscopy image of fly ash from four coal-fired thermal power plants. Due to high-temperature (>1400°C) combustion, the flux agents in the PCC fly ash melted, stuck to unmelted particles, and rounded under surface tension, forming spherical particles upon cooling. On the other hand, FBC fly ash particles have irregular shapes as they were created from a low-temperature (<900°C) combustion process and thus did not have sufficient liquid phase to form spherical particles.

**Figure 2:**
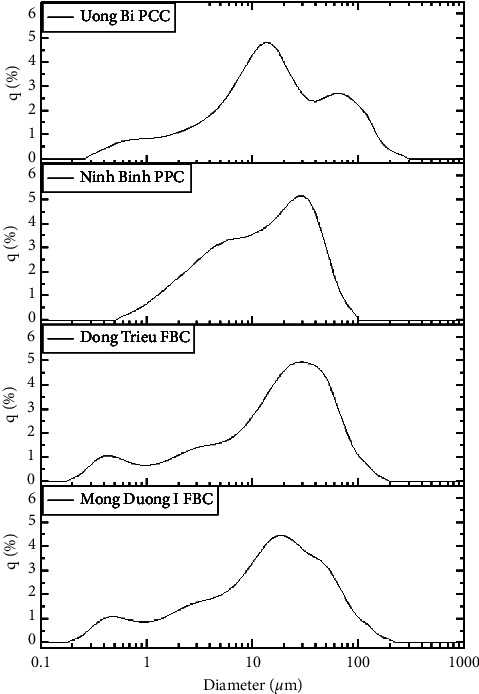
Fly ash particle size distribution.

Fly ash is a collection of fine particles that have harmful effects on human health and ecology. Significantly, the wind can blow fine fly ash particles long distances, making fly ash a global health and environmental concern [41, 42]. Most coal fly ash has particle sizes under 100 micrometers [43–45].  Figure 2 shows the particle size distribution curves of four types of fly ash. They present the frequency distribution (q in %) of particles within a specific size range. Although the particle size distribution curves are different and the combustion methods show no trend in these curves, they all have one thing in common: 90% of each sample (D90) is smaller than 100 micrometers and the median particle size (D50) is in the range of 12.4–17.6 micrometers.

The chemical composition of fly ash is quite complicated, depending on the coal origin and the combustion technique. By weight percentage, dominant in fly ash is SiO_2_, followed by Al_2_O_3_, and other oxides such as Fe_2_O_3_, CaO, K_2_O, Na_2_O, and TiO_2_ (Table 1). The introduction of limestone to the FBC boilers adds CaO to the solid combustion residues. Fly ash is also a source of heavy metals, including but not limited to V, Cr, Mn, Co, Ni, As, and Hg [46, 48, 49]. In addition, there are distinct differences between the chemical composition of different size fractions of fly ash from a boiler. Significantly higher concentrations of total carbon and heavy metals are found in smaller particles. Slightly higher concentrations of major elements, Si, Al, Fe, Ca, K Na, Mg, Mn, and Ti, are present in coarser ones [46, 48]. Fortunately, most heavy metals are locked in the glass phase of fly ash [50]. However, the ability to release heavy metals and rare Earth elements from fly ash in landfills is always a community concern. The allowable limits for the disposal of coal combustion residuals vary between countries and regions but may be tightened in the future [51, 52].

**Figure 3:**
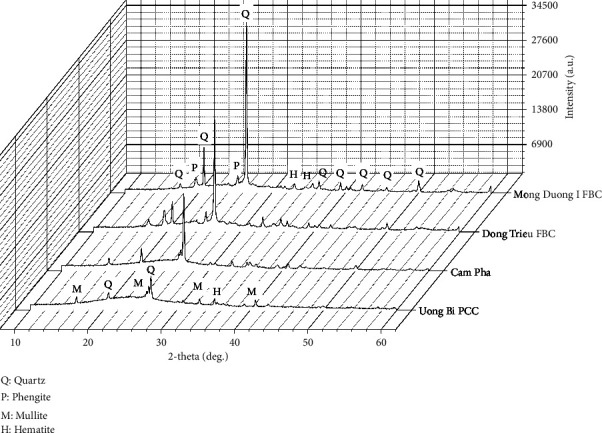
The X-ray diffraction patterns of fly ash samples.

The mineral composition of fly ash depends on the coal type and combustion techniques. X-ray diffraction (XRD) analysis of a series of fly ash showed a significant variation in the glass phase content, from 45 to 80% [53]. Besides the glass phase, quartz is the most popular crystalline phase. Its diffraction peaks stand out in the XRD patterns of all types of coal fly ash. Since PCC boilers operate at temperatures above 1400°C, high enough for mullite crystallization, its fly ash often contains mullite (3Al_2_O_3_.2SiO_2_) [54, 55]. The mineral composition of mullite is heterogeneous due to the substitution of impurities at the aluminum sites, forming solid solutions. XRD and nuclear magnetic resonance analysis on a bituminous coal fly ash showed a mullite average chemical composition of Al_4.61_Fe_0.05_Ti_0.02_Si_1.32_O_9.66_ [56]. FBC boilers operate at approximately 850°C, which is not high enough for mullite formation; thus, no diffraction peaks of mullite can be observed. Instead, hematite, phengite and anhydrite gehlenite might be present in the XRD patterns of FBC fly ash [4, 55, 57].  Figure 3 presents the XRD patterns of four types of fly ash. PCC fly ash shows only two crystal phases, quartz and mullite, while FBC fly ash shows three crystalline phases, including quartz, phengite, and hematite.

In addition to oxide solids, fly ash may contain significant amounts of unburnt carbon (UC), a result of incomplete combustion. The UC content depends on the combustion technology, operating conditions, and coal type. It is assessed by the loss-on-ignition (LOI) test [58]. Thermal analysis of fly ash confirms the presence of UC with a large exothermic peak incorporated with weight loss. The starting point of UC combustion in TG-DTA analysis varies with the type of coal fly ash [55, 59, 60]. Typically, the amount of UC in fly ash is higher than that in bottom ash [61].

Fly ash UC is an inexpensive source of activated carbon for adsorbing harmful components of flue gas such as NO_x_ [62], mercury vapor [63, 64], and organic substances [65]. However, UC is a factor hindering the use of fly ash in many construction applications. From the concrete perspective, UC adsorbs air-entraining admixture, reducing the physical properties of concrete [66]. As specified by ASTM C618 - 19 standard, the LOI content of fly ash used as pozzolanic additives in concrete should be under 6% with some tolerance to the Class F fly ash [12].

The leaching test performed on fly ash by the EN 12457–4 test method shows that the heavy metal concentrations in the leachate are below the inert waste limits specified by the European Landfill Directive [67]. However, the long-term leaching test indicates a potential risk to soil and groundwater [68]. As has been demonstrated in various studies, it is possible to immobilize heavy metals of solid wastes from wastewater treatment plants [4, 69], red sludge from bauxite processing plants [70], and blast furnace slag [71–73] in the structure of glass [74], glass ceramics [75], and ceramic materials [76]. Likewise, introducing fly ash to ceramic production is a potential solution to lock the heavy metals of fly ash in the ceramic network.

Due to high SiO_2_ and Al_2_O_3_ contents, fly ash is suitable as a precursor for aluminosilicate crystalline phases, such as mullite [77, 78] and cordierite [79–82]. In addition, the high CaO content in certain types of fly ash, especially those from FBC boilers using limestone to absorb SO_2_, makes them suitable as a raw material for glass ceramics that contain calcium silicate and calcium aluminosilicate crystalline phases [83–86]. Furthermore, due to its high glass phase content, fly ash is also considered a fluxing agent that promotes sintering in ceramics.

## 4. Firing-Associated Recycling of Fly Ash

### 4.1. Bricks

Fly ash can replace up to 80% of clay in clay bricks, but some adjustments should be made. The presence of a large amount of fly ash significantly reduces the raw mix plasticity index. Therefore, it is necessary to add plastic additives to facilitate extrusion. In addition, the porous structure of fly ash particles impedes the sintering process, increasing the porosity of the sintered product even at low degrees of replacement [87]. Hence, it may be necessary to grind the fly ash to break down its porous structure and increase the contact surface area to speed up the sintering process [37]. Along with the high porosity, the high refractoriness of fly ash requires an increase of the firing temperature by 50 to 100°C so that the physical properties of the sintered products meet those of conventional clay bricks. Most reports indicate that when using large amounts of fly ash, the firing temperature is 1050°C or higher [37, 88]. A test at the industrial scale reveals a number of obstacles that should be overcome: brick swelling and deformation due to local melting and the occurrence of black core and cracks [89].

It is possible to manufacture bricks with 100% of the solid material being fly ash. In this case, shaping requires a temporary binder because most fly ash lacks adhesive capacity. Kayali reported that although the apparent density of the fired product is 28% lower than that of conventional clay bricks, its compressive strength is 24% higher, and brick-to-mortar bond strength is 44% higher than those of the best local clay bricks [8].

It is noteworthy that heavy metals are immobilized in the ceramic structure of the brick. Sutcu et al. [88] showed that the release of heavy metals, including Cr, Mn, Ni, Cu, Zn, As, Cd, Ba, Hg, and Pb, was substantially lower than the maximum allowable limits regulated by U.S. Environmental Protection Agency (EPA) for hazardous solid waste and the threshold limits of EPA Victoria (Australia) for industrial solid waste. A study by Leiva et al. also shows that the leaching contents are below the limits of the European Landfill Directive for granular waste, the Italian national regulation for the reuse of waste in construction material, and the Dutch Soil Quality Decree for bound or shaped materials [47].

### 4.2. Ceramic Tiles

Fly ash with high flux (Fe_2_O_3_ + Na_2_O + K_2_O) contents can be used to replace feldspar in ceramic tile production. Olgun et al. were able to improve the modulus of rupture of wall tiles by replacing potassium feldspar in the raw mix with fly ash and borax solid waste without having to change the sintering temperature [90]. As the porous structure of fly ash hinders sintering, resulting in high porosity, it is mainly used as a raw material for wall tiles, equivalent to Group BIII as classified by EN 14411. This type of ceramic tile is not subject to the loading force in use but requires high water absorption for good mortar adhesion, allowing higher water absorption and lower flexural strength than those of the floor tiles [91].

Similar to that observed in clay bricks, the disruption of porous fly ash particles also improves the sintering of the ceramic tiles [92]. When high alumina fly ash is used and a floor tile is the desired product, the firing temperature must be increased to achieve the required physical properties. In particular, the sintering process becomes more difficult when the Al_2_O_3_ content is up to 40%, equivalent to fireclay refractory bricks. Wang et al. showed that for producing ceramic tiles with water absorption below 0.5% from a raw mix with 70% high alumina fly ash, the sintering temperature is as high as 1300°C [93]. Solid wastes with high flux contents, such as glass waste [94, 95], boron waste [90, 96], or lithium mine tailings, are viable additives that help lower the sintering temperature [97].

Wang et al. used high alumina content fly ash (40% Al_2_O_3_) and waste glass to make insulating ceramic tiles, consisting of a layer of foam and a layer of dense material. The raw mix of the foam layer consists of waste glass (50%), fly ash (30%), clay (15%), and feldspar (5%). The raw mix of the dense layer consists of fly ash (60–80%), clay (15%), and quartz (5–25%). With single-loading pressing and calcination at 1200°C, the foam layer performs insulation capacity with an average pore diameter of 300–500 micrometers. In addition, the bi-layer ceramic shows a flexural strength of 31.5 MPa. This way, over 70 weight percent of the tile originates from industrial waste [94].

Most fly ash has a Fe_2_O_3_ content of 4–10% (Table 1). The presence of a remarkably high Fe_2_O_3_ content gives a clay brick color to fly ash-based ceramic bodies [8]. On the other hand, the combination of Fe_2_O_3_ and UC causes the black core in bricks [98]. Therefore, if masking the ceramic body color is desired, an engobe glaze with high opacity and whiteness should be applied on the surface of the tiles before the decoration is finished.

Although a test method for determining lead and cadmium leaching from ceramic tiles is available [99], there is no harmonized requirement for hazardous element concentrations in the leachate of ceramic tiles. However, one can infer from the studies on bricks that ceramic tiles are also capable of immobilizing hazardous elements, even better than bricks because of their denser structure formed by higher sintering temperatures.

### 4.3. Insulating Materials and Lightweight Concrete Aggregates

Insulating materials and lightweight concrete aggregates feature porous structures constructed by closed pores. The sintering of these materials requires a simultaneous generation of gas and molten phases with sufficient quantity and viscosity. The molten phase entraps the gas, forming closed pores upon cooling. Fly ash is an excellent candidate for this application due to its UC and high glass phase contents. The UC oxidizes at elevated temperatures releasing carbon dioxide gas while the glass phase is ready to melt. However, if fly ash is the only raw material, the firing temperature should be up to 1300–1400°C for bloating [100]. Furthermore, UC might be low in certain fly ash sources (Table 1). Therefore, fluxes and gas-forming agents are required for (1) generating gases, (2) reducing the firing temperature, and (3) adjusting the quantity and viscosity of the liquid phase for entrapping the generated gases. The fluxes of interest include waste glass [101, 102], limestone [103, 104], and synthetic chemicals such as sodium salts [105, 106]. Some fluxes, including limestone and soda, also play the role of gas-forming agents.

Since most fly ash is not self-adhesive, a temporary binder is required to form the green pellets. On a laboratory scale, organic binders such as polyvinyl alcohol have been tested [100]. On a larger scale, clay is a popular binder due to its availability and low cost [103, 106]. Besides, bentonite [102, 104] and ordinary Portland cement [104] have been used.

### 4.4. Mullite and Cordierite Ceramics

Fly ash is also a raw material for the production of mullite ceramics due to its high SiO_2_ and Al_2_O_3_ contents. Low alumina fly ash often requires an alumina augmenting agent such as aluminum oxide [107], bauxite [77, 108], or high alumina industrial wastes [78, 109]. Unlike FBC fly ash, mullite is usually available in PPC fly ash. The presence of mullite makes the mullite crystallization in PCC fly ash-based ceramics easier than in those using FBC fly ash.

Although fluxes are available in fly ash and start forming a liquid phase at relatively low temperatures, fly ash-based mullite ceramics are often sintered at high temperatures, usually above 1400°C, because mullite is a refractory phase. Making dense mullite ceramics from PCC fly ash and bauxite, Dong et al. showed that the solid-state reaction between cristobalite and corundum occurs at temperatures below 1300°C, followed by the dissolution of the corundum into the liquid phase at higher temperatures where secondary crystallization occurs. The formation and recrystallization of mullite lead to volume expansion which is slightly dominant over the shrinkage of the sintering process. At 1600°C, the material achieves a relative density of 93.94% with spherical pores and fracture strength of 186.19 MPa [77]. Jung et al. combined aluminum oxide with PCC fly ash, from which UC was removed, to produce mullite ceramics at temperatures of 1400, 1500, and 1600°C. Although the pellet is cold isostatic pressing, the density of the fired product was relatively low (63%). This low density is due to the exaggerated grain growth of needle-shaped mullite crystals incorporated with voids formation [107].

The introduction of the supplements alters the sintering mechanism either by preventing excessive grain growth or by creating new phases, thereby improving the physical properties of the sintered products. The presence of 3Y-PSZ inhibits the crystal growth of mullite, leading to an improved fracture strength [107]. Magnesia effectively promotes sintering, significantly above 1450°C. It slightly reduces the linear thermal expansion coefficient (LTEC) at 1300°C by forming low thermal expansion *α*-cordierite. However, it slightly increases the LTEC above 1400°C due to the formation of high expansion corundum and the spinel (MgAl_2_O_4_) [108]. SiC enables the growth of mullite needle-shaped crystals out of the saturated glass phase, resulting in better bonding of the crystals, reducing the true porosity but increasing the closed-pore porosity. As a result, thermal conductivity and cold crushing strength are improved [110]. TiO_2_ hinders the sintering process at low temperatures but promotes sintering above 1300°C. This phenomenon is useful for the unsaturated sintering of porous mullite ceramic membrane supports [111].

The porous structure of fly ash particles and the recrystallization of mullite favor the fabrication of porous mullite ceramics for insulation or filtration purposes. Chen et al. fabricated porous materials from coal mine waste kaolin and spherical hollow fly ash, using bentonite as the binder and calcium iodate (Ca(IO_3_)_2_.6H_2_O) as a flocculant. After firing at 1550°C, the resulting product has a porosity of 44.73–46.12%, with 99% of the porosity being closed pores, and mullite is the main crystalline phase. The freeze-gel casting/polymer sponge technique can produce a porous mullite ceramic with a porosity of 66.1% and an average compressive strength of 45 MPa [112].

Fly ash is also suitable as a raw material for cordierite ceramics, a material well known for its low LTEC. Cordierite crystallizes in the orthorhombic system. Its dimorphism is hexagonal indialite which is more refractory than cordierite [113–115]. Although the IMA formula of cordierite is 2MgO.2Al_2_O_3_.5SiO_2_, it appears as a solid solution in practice [116, 117]. Thanks to its low LTEC, cordierite ceramic is highly resistant to thermal shock. This material also possesses high chemical resistance and a low dielectric constant. The synthesis of cordierite from fly ash requires MgO supplementing materials such as talc [118], magnesia [119], magnesite [120], or dolomite [121]. Unlike mullite, the sintering temperature of cordierite ceramics is usually below 1200°C. In addition to indialite, fly ash-based cordierite ceramics may contain mullite, cristobalite, periclase, and spinel [118, 120]. Porous cordierite ceramics from fly ash can be used for microfiltration membranes [82], catalytic substrates [118], and membrane supports [121].

### 4.5. Foam Glass and Glass Ceramics

Thanks to its high silicate glass content, fly ash can be used as a raw material for glass and glass-ceramic synthesis. Erol et al. produce glass by melting fly ash at 1500°C, glass ceramics by annealing the obtained glass at 1150°C, and ceramics by firing green pellets at 1200°C. The only phase in the glass products is amorphous, the crystalline phase in the glass-ceramic products is augite (Ca(Mg, Fe^3+^, Al) (Si, Al)_2_O_6_), and in the ceramic is quartz, mullite, and enstatite ((Mg, Fe)SiO_3_) [34]. However, making glass and glass ceramics products from fly ash can be difficult due to high SiO_2_ and Al_2_O_3_ (network formers) contents and insufficient fluxes (network modifiers). Therefore, it is necessary to add flux ingredients to lower the melting temperature.

One of the most attentive applications of fly ash-based glass is glass foam, which is considered a low-cost insulation material in construction. Besides fly ash, glass foam raw mixes often have flux and foaming agents. Most of them are industrial wastes. The commonly used flux is waste glass. It is used in large quantities comparable to fly ash [33, 122]. Alternatively, borax [122] and soda [123] can be added to the raw mix as network former and modifier, respectively. Foaming agents can be obtained from a variety of sources, including calcium carbonate [122], soda [123], and SiC [33, 124]. For instance, Bai et al. produced glass foam by melting a raw mix of fly ash, waste glass, and SiC waste at 950°C. The resulting product expanded 5.81 times compared to the green body [33].

Another fascinating application of fly ash-based glass is glass ceramics because of their high mechanical strength and thermal shock resistance. The crystalline phase in fly ash-based glass ceramics is controlled by adjusting the composition of the raw mix. The two glass ceramic systems of great interest are SiO2-Al2O3-CaO [125, 126] and SiO_2_-Al_2_O_3_-MgO [79, 127]. When a high amount of Fe_2_O_3_ is available in fly ash, the system SiO_2_-Al_2_O_3_-Fe_2_O_3_-CaO is of interest [128]. Depending on the chemical composition of the mix, the crystalline phases in the SiO_2_-Al_2_O_3_-CaO glass-ceramics include diopside (Ca(Mg, Al) (Si, Al)_2_O_6_) [125], augite (Ca(Mg, Fe)Si_2_O_6_) [34, 125], and wollastonite (CaSiO_3_) [129]. SiO_2_-Al_2_O_3_-MgO glass ceramics are of the most interest because they contain cordierite, a mineral with low LTEC, and are suitable for thermal shock applications [79, 127].

### 4.6. Portland Cement Clinker

In cement production, fly ash often serves as a pozzolanic additive, but high UC fly ash is not suitable for this application. Another possibility of using fly ash in cement production is to replace clay in the raw mixes of Portland cement clinker [130–133], belite cement clinker [134], and belite-sulfoaluminate cement clinker [135]. This perspective has been investigated from the laboratory to the industrial scale. Laboratory studies showed that the use of fly ash reduced the sintering temperature of Portland cement clinker [130, 131]. Commercial demonstration on high carbon fly ash showed that the obtained fly ash-based Portland cement performs a higher compressive strength than the normal ones et al.l ages, despite a lower fineness [132]. The use of high carbon high fly ash has the additional benefit of fuel-saving [133].

Unlike ceramic firing, SO_2_ formed in the firing process of clinker can be reabsorbed by calcium oxide. The resulting calcium sulfate helps reduce the gypsum amount required for cement setting control [136]. Therefore, using fly ash as raw material for cement clinker brings valuable economic, technical, and environmental benefits.

## 5. Challenges and Opportunities

Although it is demonstrated that fly ash-based glass and ceramic products can immobilize the heavy metals and rare elements of fly ash in their oxide networks, they are not suitable as food and drug containers. This restriction is mainly due to the stringent regulations applied to these products [137–139]. On the other hand, SO_x_ emissions from burning fly ash are also of concern because the SO_3_ content in certain types of fly ash may be high. High sulfur fly ash is often associated with FBC boilers that introduce limestone into the furnace to absorb SO_x_ gas. The SO_3_ content in fly ash can be up to 14% [140]. A dramatic increase in the sulfur dioxide content in flue gases during the firing of FBC fly ash-based ceramic tiles was observed. [92]. Unlike ceramic firing, SO_2_ formed in the firing process of cement clinker can be reabsorbed and becomes a valuable source of sulfate for cement setting control. Therefore, characterizing fly ash before recycling in these applications is critical in preventing the redistribution of toxic SO_2_ into the environment.

## 6. Conclusion

Coal fly ash is a global concern due to its small particle size and heavy metal contents with increasing emissions. Faced with concerns about wind-blowing dust and heavy metal leakage from fly ash landfills and storage sites, efforts to use fly ash for safe and value-added applications are underway. Notable among them are firing-associated measures, including the production of ceramics, lightweight concrete aggregates, glass, and glass ceramics. There is almost no restriction for fly ash in these applications. Most notably, the recycling of fly ash and other industrial solid wastes can be combined in these applications without violating regulations on heavy metals released during use. Besides, the unburnt carbon content can alleviate the heat required for firing. The firing temperature, product phase composition, and physical properties of the products can be controlled by supplementing agents. However, the sulfur content might be high in certain types of fly ash and go into the flue gas as sulfur dioxide. This problem can be solved in cement kilns but should be addressed in other applications.

## Figures and Tables

**Table 1:** Chemical composition of fly ash.

Chemical composition	Ninh Binh, Vietnam^a^	Mong Duong I, Vietnam^a^	Seyitomer, Turkey [7]	Tennessee, US [15]	XianYang, China [33]	Bokaro, India^b,d^ [46]	Bokaro, India^c,d^ [46]	Spain^d^ [47]
SiO_2_ (wt.%)	45.20	52.16	57.21	49.54	54.39	39.57	51.94	45.02
Al_2_O_3_ (wt.%)	19.99	23.65	20.39	19.11	23.21	22.13	26.41	25.18
Fe_2_O_3_ (wt.%)	6.86	5.97	10.89	14.79	11.50	3.21	5.21	16.52
CaO (wt.%)	1.52	1.94	2.75	6.23	4.77	0.42	0.49	8.53
MgO (wt.%)	1.29	1.09	4.96	0.42	1.73	0.19	0.27	0.97
K_2_O (wt.%)	3.13	3.30	1.36	—	1.32	0.93	0.95	2.14
Na_2_O (wt.%)	0.32	0.19	0.40	—	0.37	0.13	0.16	0.43
TiO_2_ (wt.%)	0.57	0.91	0.81	—	—	2.08	2.09	—
SO_3_	—	—	—	0.34	—	1.45	0.50	—
LOI (wt.%)	20.15	9.46	0.94	2.43	13.38	29.8	9.67	0.7
Cd (ppm)	—	—	—	—	—	—	—	0.9
Pb (ppm)	—	—	79.0	—	—	31.06	63.42	58
Zn (ppm)	—	—	112.6	—	—	47.2	76.6	309
Cu (ppm)	—	—	98.8	—	—	73.8	97.7	-
Cr (ppm)	—	—	454.5	—	—	140.7	137.4	149
Ni (ppm)	—	—	1975.9	—	—	77.5	65.7	74
Mn (ppm)	—	—	790.4	—	—	117.8	268.4	-

^a^ Present work, by chemical analysis. ^b^ Fraction retained by Sieve No. 150 BS (+104 *μ*m). ^c^ Fraction passed through by Sieve No. 300 B.S and retained by Sieve No 350 BS (−53 *μ*m to + 45 *μ*m). ^d^ Incomplete list of the analyzed heavy metals and rear Earth elements from the cited sources.

## Data Availability

All data generated and analyzed to support the findings of this study are available within the article.
